# The autophagy inhibitor spautin-1, either alone or combined with doxorubicin, decreases cell survival and colony formation in canine appendicular osteosarcoma cells

**DOI:** 10.1371/journal.pone.0206427

**Published:** 2018-10-29

**Authors:** Courtney R. Schott, Latasha Ludwig, Anthony J. Mutsaers, Robert A. Foster, Geoffrey A. Wood

**Affiliations:** 1 Department of Pathobiology, Ontario Veterinary College, University of Guelph, Guelph, Ontario, Canada; 2 Department of Clinical Studies, Ontario Veterinary College, University of Guelph, Guelph, Ontario, Canada; 3 Department of Biomedical Sciences, Ontario Veterinary College, University of Guelph, Guelph, Ontario, Canada; Universite de Nantes, FRANCE

## Abstract

Dogs diagnosed with appendicular osteosarcoma typically succumb to metastatic disease within a year of diagnosis. The current standard of care for curative intent, amputation followed by adjuvant chemotherapy, increases survival time but chemoresistance is a major contributor to mortality. Unfortunately, the mechanisms driving the progression of metastatic disease and the development of chemoresistance are unknown. One theory is that autophagy may contribute to chemoresistance by providing neoplastic cells with a mechanism to survive chemotherapy treatment. Our objective was to evaluate the effect of combining an autophagy inhibitor with a standard chemotherapeutic drug on response to chemotherapy in canine appendicular osteosarcoma cells. We hypothesized that combining the autophagy inhibitor spautin-1 with doxorubicin treatment would enhance chemoresponsiveness. Using commercial (D17) and primary cell lines derived from 1° and 2° sites of osteosarcoma, we showed that this combination treatment enhances cell killing and inhibits colony formation. Our findings support the theory that autophagy contributes to chemoresistance in canine appendicular osteosarcoma and indicate that adding an autophagy inhibitor to the standard of care has the potential to improve outcome.

## Introduction

Despite being the most common and aggressive bone neoplasm of dogs, the treatment used for canine appendicular osteosarcoma has been largely unchanged for decades [1]. The addition of adjuvant chemotherapy post-amputation was investigated in the late 1980s [2–6], was further evaluated and made common practice in the 1990s [3,7–10], and remains the standard of care for curative intent today. Unfortunately, even with aggressive chemotherapy post-amputation, most dogs succumb to metastatic disease less than a year after diagnosis [11].

Multiple attempts have been made to extend survival time by altering the current standard of care for curative intent, as well as to improve the efficacy of treatment against metastatic disease, but canine osteosarcoma is highly chemoresistant. Alternating doses of the most commonly used chemotherapeutics, doxorubicin and carboplatin, does not improve survival time, but may reduce adverse effects [12–18]. The use of these two chemotherapeutics has also been compared retrospectively with no significant difference in outcome [19]. New or alternative therapeutic agents, including other platinum compounds, different classes of chemotherapeutics, bisphosphonates and other palliative therapies, liposome-encapsulated drugs, matrix metalloproteinase inhibitors, mTOR inhibitors, tyrosine kinase inhibitors, human cytotoxic T-cells, immunotherapies, drugs that target multi-drug resistance, and even personalized strategies have not proved superior to the current regimen [1,20–32]. There is however, a new vaccine with promising phase I results [33]. Neoadjuvant chemotherapy is a component of the standard of care for the treatment of human conventional osteosarcoma, the human equivalent of canine appendicular osteosarcoma. However, there is currently no evidence that neoadjuvant treatment improves outcome in dogs with appendicular osteosarcoma [34]. Additional *in vivo* studies in dogs have investigated the treatment of metastatic disease after chemotherapy fails, but to date no significant improvements in survival time have been gained [18,24,35,36]. Chemoresistance is therefore a major hurdle in the management of canine appendicular osteosarcoma, since metastatic disease develops despite aggressive chemotherapy. The search continues for treatments that can prolong life and prevent progression in dogs diagnosed with appendicular osteosarcoma.

Autophagy is a self-digestion and recycling mechanism used by cells to survive harsh environmental conditions. Autophagy and/or autophagy dysregulation are suspected to play various, sometimes conflicting roles in the development and progression of neoplasia [37–39]. One hypothesis is that neoplastic cells use autophagy to survive treatment with chemotherapeutic agents. Blocking autophagy under these circumstances could trigger apoptosis, enhancing the effects of the chemotherapeutic agent [40].

Spautin-1 (specific and potent autophagy inhibitor-1) is a small molecule that inhibits autophagy by enhancing degradation of beclin-1, a protein required for the initiation of autophagy [41]. Since its discovery in 2011, multiple *in vitro* studies confirm that this drug can inhibit autophagy, induce cancer cell death, and enhance the effectiveness of radiation therapy and chemotherapy in multiple human cancer cell lines, including a human osteosarcoma cell line [40,42–47]. The chemotherapeutic agent doxorubicin induces autophagy in human osteosarcoma cell lines [48]. We hypothesized that inhibition of autophagy would render canine osteosarcoma cells more sensitive to chemotherapy. Our objective was to evaluate the effect of pre-treatment with the autophagy inhibitor spautin-1 on the response of canine osteosarcoma cells to doxorubicin.

## Materials and methods

### Cell lines and cell maintenance

Both primary and commercial cells lines were used. The primary cell lines were derived in-house from 1° and 2° tumours removed from dogs with appendicular osteosarcoma. For most experiments, there were 6 canine cell lines used, 2 of each representing: non-cancer cells [MDCK (Madin Darby Canine Kidney) and OVC-cMES-103], cells derived from 1° osteosarcoma (OVC-cOSA-75 and OVC-cOSA-106), and cells derived from 2° osteosarcoma (D17 and OVC-cOSA-31). The commercial cells lines, D17 and MDCK, were obtained from the American Type Culture Collection (ATCC; S1 Table). The primary cell lines were derived in our laboratory either by the current author (CS), OVC-cMES-103 and OVC-cOSA-106, or by previous members of the laboratory. All cells were maintained at 37.0°C, 5% CO_2_ (Thermo Fisher Scientific, HERAcell 150i CO_2_ Incubator) in 10 cm plates (Corning, 430167) with Dulbecco′s Modified Eagle′s Medium (HyClone DMEM High Glucose, SH30022.01) with L-glutamine (Corning, 25-005-Cl) and 10% fetal bovine serum (FBS; Gibco, 12483–020), hereafter called standard media.

#### Derivation of primary cell lines

All animal experiments were performed according to Canadian Council of Animal Care guidelines. This study was approved by the Animal Care Committee of the University of Guelph and consent to collect tissue samples was obtained from the dogs’ owners. For derivation of cell lines OVC-cMES-103 and OVC-cOSA-106, tissue samples were collected from 2 dogs (see S1 Table for animal details) that underwent amputation at the Ontario Veterinary College (OVC) Health Sciences Centre (HSC) as a component of the standard of care for appendicular osteosarcoma. Sterile dissection instruments were used to extract tissue. Samples were dipped in 70% ethanol, rinsed with phosphate buffered saline (PBS), and then submerged in sterile PBS or standard media for 1–2 hours at 4°C in 50 mL tubes (Fisher Scientific, 06-443-18).

Sterile procedures were used to transfer the tissue samples into 10 cm culture plates. Tissues were manually minced with a scalpel blade into 1–2 mm pieces and covered with 10 mL of standard media plus 10 μg/mL gentamicin (Sigma, G1272-100ML). These plates were then transferred into the incubator for a minimum of 21 days at 37.0°C, 5% CO_2_. Once the cells reached confluency, they were rinsed with 2 mL of PBS, trypsinized with 1 mL of 0.05% Trypsin with 0.53 mM EDTA (Corning, 25-052-Cl), resuspended in media, and split into multiple 10 cm plates. Cells were continually passaged as plates reached confluency and gentamicin was eventually eliminated from the media, prior to freezing or experimental use.

Cells from various passages were frozen and stored in liquid nitrogen for future use. First, they were resuspended in standard media plus an additional 10% FBS and 10% DMSO (dimethyl sulfoxide; Fisher Scientific, BP231-100). Resuspended cells were aliquoted into 1.2 mL cryogenic vials (Corning, 430487). Prior to being transferred into liquid nitrogen, vials were immersed in isopropyl alcohol and placed in a -80°C freezer for 24 hours. Individual vials of cells were thawed as necessary. The other primary cell lines, OVC-cOSA-75 and OVC-cOSA-31, were previously derived in-house using similar methods.

#### Colony formation in soft agar

For the base layer, 1.5 mL of molten 0.8% agarose (UltraPure LMP Agarose; Invitrogen, 16520–100) was added to each well of a 6-well plate (Costar, 3506) and was left to solidify at room temperature for at least 1 hour. Confluent cells were trypsinized and resuspended in standard media. Resuspended cells were mixed 1:1 with 0.4% Trypan Blue (Gibco, 15250–06) and live cells were counted using the TC20 Automated Cell Counter (BioRad, Serial No. 508BR02851). Cells were resuspended in 0.4% agarose (5000 cells/1.5 mL) at 37.0°C and 1.5 mL/well was added on top of the base layer. The plates were left to solidify in the biological safety cabinet for 1 hour prior to being moved to the incubator. An entire 6-well plate was used for each cell line. After 28 days, colonies were stained and fixed with 0.05% crystal violet (Fisher Scientific, C581-25) containing 3% formalin (Surgipath 10% neutral buffered formalin; Leica, 3800780) for 15 minutes. Plates were then washed with PBS and after drying, the number of colonies per well was quantified using an Olympus SZX7 microscope. Cell lines that did not form colonies in soft agar were designated as non-cancer cells.

### Protein collection and western blot

Cells were maintained in standard media at 37.0°C, 5% CO_2_. For each cell line, the seeding density necessary to reach 90–100% confluency at 72 hours was determined for 6-well plates under untreated conditions. For each experimental replicate for each cell line, cells for all treatment conditions (see Treatment subsection) were plated in duplicate wells. In half of these wells, 6 hours prior to the endpoint, 75 μM per well of hydroxychloroquine sulfate (HCQ; Sigma, H0915-5MG Lot# 046M4758V) was added to inhibit lysosome fusion with the autophagosome, thus preventing degradation of the proteins required to evaluate autophagic carrier flux via western blot analysis. HCQ treated proteins were used for the LC3 and P62 western blots. Optimization experiments where protein was collected for LC3 and P62 western blots in the absence of HCQ were also performed.

At the endpoint, 72 hours after seeding, the media was aspirated and cells were rinsed with cold PBS, then the plates were transferred onto ice. Either 60 or 120 μL/well, depending on cell viability, of RIPA buffer (Sigma Life Science, R0278-50ML) was applied to the cells for 1 minute, then cells were harvested using a cell scraper (25 cm Sterile Disposable Cell Scraper; Fisher Scientific, 08-100-241). Cell lysate from each well was transferred into separate 1.5 mL tubes (Fisher Scientific, 05-408-129) and was quickly vortexed. Lysate remained on ice for 30 minutes, being vortexed every 2–3 minutes. The samples were centrifuged at 16,000 x g for 30 minutes at 4°C before removing the supernatant and transferring it to new 1.5 mL tubes. If not being used immediately, proteins were stored at -80°C. Protein concentration was determined using a Bradford assay (Bio-rad Protein Assay Dye Reagent Concentrate, 500–0006).

Individual proteins were combined 1:1 with 2x reducing buffer [5 mL Laemmli Sample Buffer (BioRad, 161–0737) and 270 mg Dithiothreitol (BioRad, 161–0611)] in preparation for loading, then maintained at 95°C for 5 minutes in a heating block. Experimental protein samples (20 μg) and PiNK Plus Prestained Protein Ladder (GeneDireX Inc., PM0005) were loaded into separate wells of 12% TGX Stain-Free FastCast Acrylamide gels (BioRad, 1610185). Gels were submerged in 1x Tris-Glycine SDS running buffer (diluted from 10x; BioRad, 1610732) and proteins were separated by molecular weight at 200 V for up to 45 minutes. Proteins were transferred from the gel to a Trans-Blot Turbo Mini-size PVDF membrane (BioRad, 1704272) using the Trans-Blot Turbo Transfer System (BioRad, 1704150) at 25 V for 3 minutes. The membrane was then washed twice while being gently agitated in tris-buffered saline (TBS) containing 0.5% Tween 20 (MP Biomedicals, 194841; TBST). The membrane was blocked with 5% skim milk in TBST for 1 hour at room temperature with motion. Primary antibody was diluted (Table 1) with 5% skim milk in TBST and incubated for 14–16 hours at 4°C on a rocker. The membrane was then washed twice with TBST. Secondary antibody diluted with 5% skim milk in TBST (Table 1) was applied to the membrane for 1 hour at room temperature with motion, prior to being washed twice with TBST and once with TBS. Clarity Western ECL Substrate (BioRad, 1705060), 10 mL/membrane, was applied for 5 minutes before the chemiluminescence signal was detected by the ChemiDoc XRS (BioRad, 1708265).

**Table 1:** Antibodies used for western blot analysis.

	Primary Antibody				Secondary Antibody			
Protein Target	Species	Company	Product #, Lot#	Dilution	Species	Company	Product #, Lot#	Dilution
**Beclin-1**	Mouse	LSBio	LS-C172820, 66461	1:2500	Goat	Thermo Fisher Scientific	G21040	1:2000
**P62**	Rabbit	MBL	PM045, 018	1:10000	Goat	Thermo Fisher Scientific	G-21234, 1419716	1:10000
**LC3**	Rabbit	Cell Signaling	PM036, 029	1:10000	Goat	Thermo Fisher Scientific	G-21234, 1419716	1:10000
**pS6RP**	Rabbit	Cell Signaling	4857, 2	1:10000	Goat	Thermo Fisher Scientific	G-21234, 1419716	1:5000
**Beta actin**	Goat	Santa Cruz Biotechnology	Sc-1616, F1213	1:10000	Rabbit	Thermo Fisher Scientific	611620, 1305431A	1:2500

P62, sequestosome 1; LC3, microtubule-associated protein light chain 3; pS6RP, phospho-S6 ribosomal protein

### Cell viability assays

For each cell line, the seeding density determined for the 6-well plates (described previously in the Protein Collection and Western Blot subsection), was adjusted to the surface area of a 96-well plate (Corning Costar, 3997). Cells were exposed to various conditions (see Treatment subsection) and 6 hours prior to treatment endpoint 11 μL of resazurin (R&D Systems, AR002) was added to each well. Fluorescence was measured at 525/590 nm with a BioTek Synergy HT plate reader using Gen5 version 1.11 software (BioTek) and values were normalized to the mean of the cell-free media-only wells. The values of the non-treated control wells were assigned 100% viability and cell viability for the other conditions was calculated accordingly. For each experiment, 6 plates were used with 36 wells per condition and the experiment was performed twice for each cell line (n = 72 wells for each condition of each cell line). Repetitions were combined for statistical analysis.

### Clonogenic assays

Confluent cells were trypsinized and resuspended in standard media to create a single cell suspension. The cell density of the solution was measured using the TC20 Automated Cell Counter. The cell suspension was further diluted with standard media to reach the appropriate seeding density. For each cell line, the seeding density necessary for non-treated cells to reach approximately 50–100 colonies per well in 7–9 days was established; to meet the definition of a colony, a minimum of 50 cells was required. After seeding, the cells were left to attach for 24 hours prior to being treated with various conditions (see Treatment subsection). Under all conditions, 72 hours after seeding, treated media was removed and cells were covered with fresh media before being returned to the incubator. At the endpoint, 7–9 days post-seeding, cells were rinsed twice with PBS then 1 mL of 0.01% crystal violet in 10% formalin was applied to the cells for 15 minutes. The fixative was removed, and cells were rinsed at least twice with PBS, and if necessary submerged in water until excess stain was removed. The number of colonies per well was counted and a minimum of 20 colonies per well was required. Each treatment condition was represented by 3–6 wells. Plating efficiency (PE) was calculated based on the colonies formed in the non-treated wells and was subsequently used to calculate the surviving fraction (SF) for the treated wells. For each cell line, the experiment was performed twice (n = 6–12 wells for each condition of each cell line).

### Treatment conditions

The drugs used for the various treatments were spautin-1 (Sigma, SML0440) and a chemotherapeutic agent (doxorubicin hydrochloride; Pfizer DIN 02410397). Doxorubicin IC50s were determined for cell viability (96-well plates, 18 wells/dose), using R statistical analysis software [49], and for colony formation (6-well plates, 2–4 wells/dose). Similarly, determination of the spautin-1 IC50 was attempted for each cell line for the cell viability experiments. For the IC50s calculated using R statistical analysis software, the “ic50” package [50] was used, and the goal was to attain a coefficient of variation of < 0.05 and a maximum standard deviation of ≤ 0.2 [51].

Cells were exposed to both single-agent and combination treatments using both high and low doses of each drug. For the various conditions, cells were exposed to each individual drug for 24 hours at a time. Pre-treatment with spautin-1, followed by doxorubicin defines the combination treatment. All drugs were diluted to the appropriate concentration in standard media.

Cells used for viability assays and western blot were seeded in their respective 96- and 6-well plates simultaneously from the same single cell suspension (i.e. experiments were run in parallel). At 24 hours post-seeding, spautin-1 was applied to the appropriate wells and fresh media was applied to the cells not receiving pre-treatment. At 48 hours post-seeding, the treated and non-treated media was removed from all wells and either doxorubicin or fresh media was added to the wells depending on the condition. At 68 hours post-seeding, 11 μL of resazurin was added to each well of the 96-well plates for the cell viability assays and 75 μM of HCQ was added to half of the wells for western blot. At 72 hours, fluorescence was determined for the viability assays and protein was collected for western blot analysis.

### Statistical analysis

R statistical analysis software was used to perform all analyses [49]. The Shapiro-Wilk test was used to test for normality. For normally distributed data, the Bartlett test was used to test the assumption of homogeneity of variance, while for non-normal data the “car” package was used to perform the Levene test [52].

For normal/non-normal data where groups had equal variances, one-way ANOVA followed by Tukey’s Honestly Significant Difference test was performed using the “multcomp” package [53]. For normal/non-normal data where groups had unequal variances, Welch’s ANOVA followed by the Games-Howell posthoc test was performed using the “onewaytests” and “userfriendlyscience” packages respectively [54,55]. When *p* < 0.05, differences were considered significant.

## Results

### Derivation, seeding densities, and IC50s

All osteosarcoma cell lines, both commercial (D17) and those derived in our lab, were adherent in culture, had a spindle-cell morphology, and formed colonies in soft agar, suggesting a neoplastic origin. OVC-cMES-103 cells also had a spindle-cell morphology but did not form colonies and are therefore presumed to have a non-cancerous mesenchymal origin. The 72 hour seeding densities for each cell line in 96- and 6-well plates, used for cell viability assays and protein collection, are provided in S2 Table. The seeding densities used for the clonogenic assays varied by treatment and are also provided in S2 Table. Non-cancer cell line OVC-cMES-103 did not form colonies within 8 days.

Cells varied in their susceptibility to doxorubicin, with the non-cancer cell lines being least susceptible, and the cell lines derived from 2° osteosarcoma being most susceptible (S1 Fig and S2 Table). The standard deviation was ≤ 0.02 for all cell lines and the coefficient of variations was < 0.05 for all cell lines except MDCK. For the cell viability assays, doxorubicin IC50 was designated as the high dose and for the low dose, the IC50 was divided by 20. Only one dose, the IC50, was used in the colony formation assays (S2 Fig and S2 Table).

During spautin-1 IC50 determination, drug precipitation occurred at concentrations > 100 μM. Most cell lines were minimally affected by spautin-1, and IC50 determination was only possible for a single cell line (2° osteosarcoma, D17; S3 Fig), but was not considered reliable because the IC50 occurred at a dose > 100 μM. Subsequently, 100 μM was designated as the high dose of spautin-1 for all cell lines and experiments. In the most severely affected cell line, D17, 5 μM was the lowest dose where an effect on cell viability was seen (S3 Fig), and therefore 5 μM was selected as the low dose of spautin-1 for all cell lines and experiments.

### Cell viability assays

**Fig 1:**
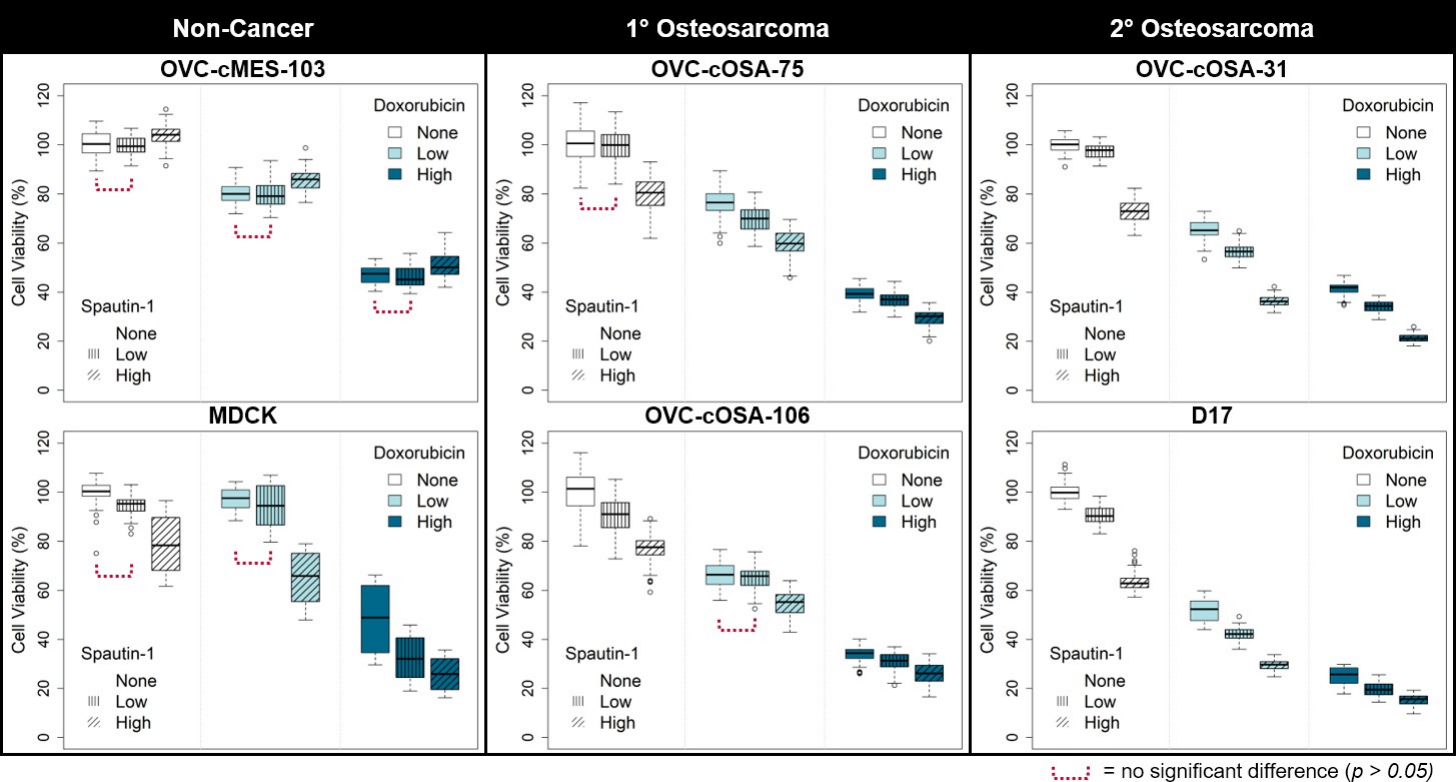
Cell viability of canine cells treated with the autophagy inhibitor spautin-1, doxorubicin, or a combination, with spautin-1 applied pre-chemotherapy. Box plots representing cell viability measured using a resazurin assay for canine cell lines treated with spautin-1 (Low = 5 μM, High = 100 μM) or doxorubicin (Low = IC50/20, High = IC50) for 24 hours each as single-agent or combination treatments, using both low and high doses. Using Welch’s ANOVA followed by the Games-Howell test, differences between all treatments were statistically significant (*p <* 0.05), except where indicated (n = 72 wells per box).

For all cell lines the treatment groups had non-equal variances, thus requiring Welch’s ANOVA. The cell viability results are graphically depicted in Fig 1. For all cell lines except MDCK, the difference between replicate treatments varied by ≤ 7% (Fig 1). For MDCK, the difference in the effect caused by the same treatment between replicate wells varied by up to 28% (Fig 1).

#### Single-agent treatment

Both low and high doses of doxorubicin consistently reduced cell viability in all cell lines by up to 52% and 38–78%, respectively (*p* < 0.05, Fig 1). Low dose spautin-1 reduced cell viability in all cell lines by up to 11% (Fig 1); in both non-cancer cell lines, this was not significantly different from no treatment, but it was statistically significant in 3 of 4 cell lines derived from osteosarcoma (*p* < 0.05 for OVC-cOSA-106, OVC-cOSA-31, and D17). High dose spautin-1 significantly reduced cell viability in all cell lines, except OVC-cMES-103, by 12–38% (*p* < 0.05); in OVC-cMES-103, a non-cancer mesenchymal cell line, high dose spautin-1 significantly increased viability by 3–5% (*p* < 0.05; Fig 1). Cell lines derived from 2° osteosarcoma were most sensitive to high dose spautin-1, where it decreased cell viability by 27–38% (*p* < 0.05; Fig 1).

#### Combination treatment

Low dose spautin-1 applied prior to chemotherapy reduced cell viability compared to low and high dose doxorubicin alone by up to 12% (*p* < 0.05 for OVC-cOSA-75, OVC-cOSA-31, and D17) and by up to 8% (*p* < 0.05 for all but OVC-cMES-103), respectively (Fig 1). Pre-chemotherapy high dose spautin-1 reduced cell viability compared to low and high dose doxorubicin alone by an additional 10–30% and 6–20%, respectively (*p* < 0.05; Fig 1). In the cell lines derived from osteosarcoma, the effect of the pre-treatment became less evident with increasing doxorubicin (Fig 1). The combination effect was never more than the sum of the effects caused by each drug given alone.

### Clonogenic assays

For all cell lines except D17, the data was normally distributed. All cell lines except MDCK met the homogeneity of variance assumption. The standard error within treatment groups across all cell lines ranged from 0.96–6.94% and was typically highest for low dose spautin-1 alone.

#### Single-agent treatment

**Fig 2:**
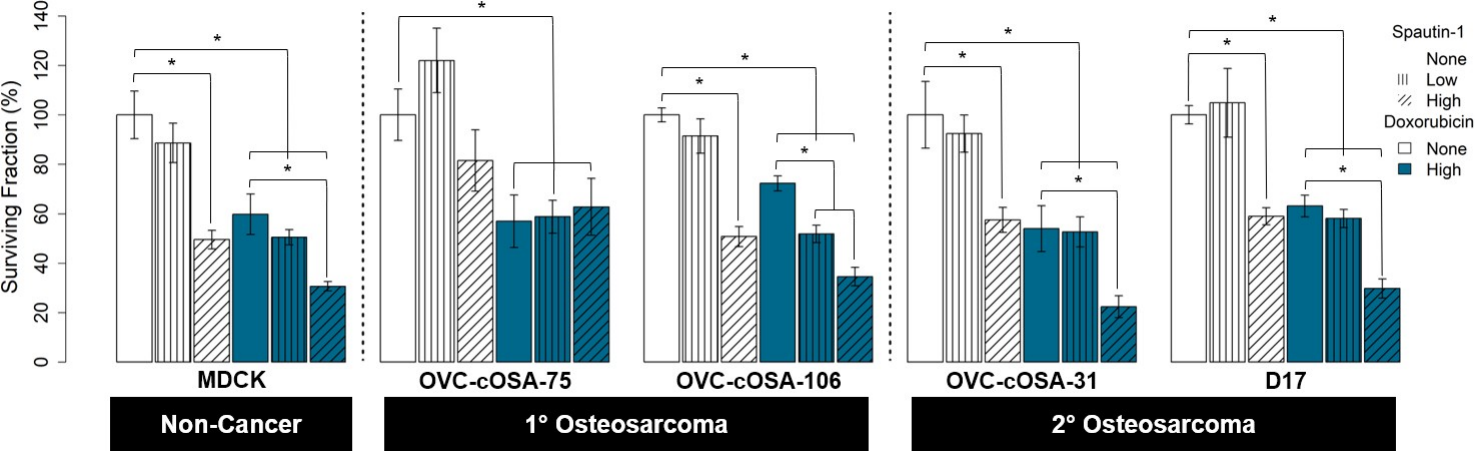
Colony formation in canine cells treated with the autophagy inhibitor spautin-1, doxorubicin, or a combination, with spautin-1 applied pre-chemotherapy. Bar graphs representing the mean surviving fraction of colonies determined using a clonogenic assay for canine cell lines treated with spautin-1 or doxorubicin for 24 hours each as single-agent or combination treatments, using both low (5 μM) and high (100 μM) doses of spautin-1. One-way ANOVA followed by Tukey’s Honestly Significant Difference test used for statistical analysis (* = *p <* 0.05, n = 6–12 wells per bar). Error bars represent standard error.

Doxorubicin significantly reduced colony formation for all cell lines by 27–52% (*p* < 0.05; Fig 2). Low dose spautin-1 was not significantly different from no treatment in any cell line, but either mildly reduced colony formation (4–18%) or enhanced it by 2–30% (Fig 2). High dose spautin-1 significantly and consistently reduced colony formation by 13–53% (*p* < 0.05) in all cell lines except OVC-cOSA-75; this reduction was greater than that of doxorubicin alone for 2 of 4 osteosarcoma cell lines (Fig 2).

#### Combination treatment

For all cell lines, except OVC-cOSA-75, pre-treatment with low or high dose spautin-1 further reduced colony formation compared to doxorubicin alone (Fig 2). The difference compared to doxorubicin alone when low dose spautin-1 pre-treatment was added was statistically significant only for OVC-cOSA-106 (*p* < 0.05), while the addition of high dose spautin-1 pre-treatment compared to doxorubicin alone was significant in all cell lines (*p* < 0.05), except OVC-cOSA-75. For OVC-cOSA-106, the effect of pre-treatment with low dose spautin-1 combined with doxorubicin was more than the sum of the effects of the individual drugs given alone (Fig 2).

### Western blot

**Fig 3:**
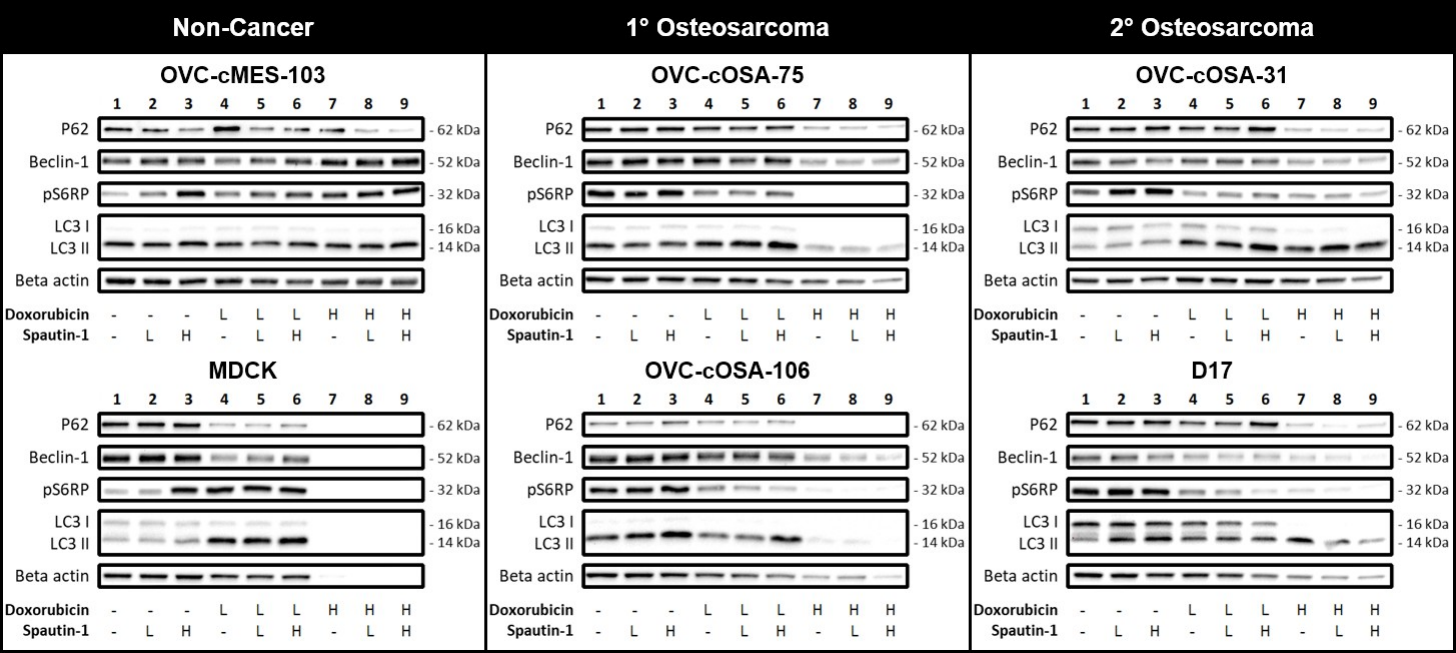
Expression of autophagy related proteins in canine cells treated with the autophagy inhibitor spautin-1, doxorubicin, or a combination, with spautin-1 applied pre-chemotherapy. Western blot expression of beclin-1, P62, pS6RP (phospho-S6 ribosomal protein), and LC3 (microtubule‑associated protein light chain 3) in canine cell lines treated with spautin-1 (Low = 5 μM, High = 100 μM) or doxorubicin (Low = IC50/20, High = IC50) for 24 hours each as single-agent or combination treatments, using both low and high doses. Beta actin was used as a loading control.

All western blots are displayed in Fig 3. In general, the intensity of the beta actin bands was reduced with increasing doxorubicin, despite equal loading of total protein (Fig 3). For MDCK, most bands representing cells treated with high dose doxorubicin were not detectable (Fig 3). Therefore, the bands from MDCK cells treated with high dose doxorubicin were not examined for trends. Unlike the others, protein levels in OVC-cMES-103 and OVC-cOSA-31 were stable, even after high dose doxorubicin treatment, based on the loading control. For the autophagy related proteins in most osteosarcoma cell lines treated with high dose doxorubicin, band intensity was reduced, and sometimes lost, beyond what would be expected based on the beta actin bands.

#### Single-agent treatment

Chemotherapy is known to induce autophagy [56], thus beclin-1 would be expected to increase in response to treatment with increasing concentration of chemotherapy. This was observed in OVC-cMES-103 cells (non-cancer mesenchymal cells) but was not seen in any in cell lines derived from osteosarcoma; instead, beclin-1 expression typically decreased with increasing doxorubicin (lanes 1 vs 4 vs 7; Fig 3). As a beclin-1 inhibitor, spautin-1 is expected to reduce beclin-1 expression because it enhances its degradation. This expected trend of decreasing beclin-1 expression was observed in the 2° osteosarcoma cell lines, but spautin-1 had no effect on beclin-1 expression in the non-cancer and 1° osteosarcoma cell lines (1 vs 2 vs 3; Fig 3).

In most cell lines, autophagic carrier flux was induced by low dose doxorubicin, based on an increase in LC3II expression, as expected (lanes 1 vs 4; Fig 3), while there was no change for OVC-cOSA-106 and OVC-cMES-103. In OVC-cMES-103, a non-cancer cell line, and the 2° osteosarcoma cell lines, LC3II expression was either maintained or further intensified with high dose doxorubicin, as expected (lanes 4 vs 7; Fig 3). However, this was not the case for our 1° osteosarcoma cell lines, which showed a marked reduction in LC3II expression after treatment with high dose doxorubicin (lanes 1 vs 4 vs 7; Fig 3). Also, unexpectedly, in all cell lines, LC3II expression either did not change or actually increased with increasing spautin-1 (lanes 1 vs 2 vs 3; Fig 3); as an autophagy inhibitor, spautin-1 is expected to reduce LC3II expression. Optimization experiments in the absence of HCQ with D17 cells indicate that both spautin-1 and doxorubicin alter complete autophagic flux, and not just autophagic carrier flux (S4 Fig).

P62 is depleted during autophagy. P62 band intensity typically decreased with increasing doxorubicin (lanes 1 vs 4 vs 7; Fig 3); this occurred in the presence and absence of HCQ (lanes 1 vs 4 and 7 vs 10; S4 Fig), indicating increased autophagic flux. In the cell lines derived from osteosarcoma, P62 expression tended to remain fairly constant or to subtly increase with increasing spautin-1 (lanes 1 vs 2 vs 3; Fig 3), as expected. Unexpectedly, treatment with increasing spautin-1 concentration in the non-cancer cell lines resulted in decreasing P62 intensity.

In the non-cancer cell lines, pS6RP expression increased with increasing doxorubicin, while the opposite was observed in all osteosarcoma lines (lanes 1 vs 4 vs 7 if applicable; Fig 3). pS6RP expression generally increased with increasing spautin-1 (lanes 1 vs 2 vs 3; Fig 3), which is a logical pattern if autophagy is being inhibited.

#### Combination treatment

When considering the effects of combination treatment, one must keep in mind that in most cell lines high dose doxorubicin diminished the expression of the loading control. This decreased expression was observed despite both the presence of live cells reflected in the cell viability results during IC50 determination (S1 Fig) and equal protein loading. The most common observation across all cell lines was that the effect of increasing spautin-1 on autophagy related protein expression was either greatly reduced or completely lost in the presence of high dose doxorubicin (lanes 1 vs 2 vs 3, 4 vs 5 vs 6, and 7 vs 8 vs 9); this was true for P62 expression in all osteosarcoma cell lines (Fig 3). Occasionally, the expression trend seen with increasing spautin-1 in the absence of doxorubicin (lanes 1 vs 2 vs 3) and in combination with low dose doxorubicin (lanes 4 vs 5 vs 6) was exactly the opposite to the trend seen with increasing spautin-1 in combination with high dose doxorubicin (lanes 7 vs 8 vs 9); this was true for LC3II in D17, beclin-1 in OVC-cOSA-106, and pS6RP in OVC-cOSA-31 (Fig 3).

## Discussion

Our investigation evaluated the effect of pre-treatment with the autophagy inhibitor spautin-1 on the response of canine osteosarcoma cells to doxorubicin with respect to cell viability, colony formation, and expression of autophagy related proteins. We hypothesized that doxorubicin would induce autophagy in canine osteosarcoma cells and that the concomitant autophagy inhibition induced by spautin-1 would render these cells more sensitive to chemotherapy. Consistent with our hypothesis, spautin-1 treatment prior to doxorubicin treatment resulted in a reduction in both cell survival and colony formation compared to doxorubicin alone in the D17 commercial cell line and all of our primary cell lines derived from canine osteosarcoma. The effect on cell viability was never more than the sum of the effects of the individual drugs given alone, but for colony formation in a single cell line derived from 1° osteosarcoma, the effect was greater than this sum. When used in combination, spautin-1’s ability to enhance cell killing diminished with increasing concentration of doxorubicin, but the overall trend of enhanced killing was consistent.

A study using triple negative breast cancer cells found that 10 μM of spautin-1 for 24 hours (our low dose was 5 μM for 24 hours) had minimal effect on colony formation, but when used in combination with a chemotherapy drug, paclitaxel, it significantly reduced colony formation, even in paclitaxel-resistant cells [42]. In our canine osteosarcoma cells, the reduction in colony formation induced by high dose spautin-1 alone was equivalent to, or even greater than, that of doxorubicin alone (Fig 2). These findings are significant to canine osteosarcoma because the disseminated neoplastic cells that remain after amputation and survive chemotherapy to form new colonies are responsible for metastatic disease, which is the most common cause of mortality. A new therapy that can limit colony formation at a level that is similar or superior to the current standard of care has great potential to improve the overall outcome for canine osteosarcoma and may have potential applications to human osteosarcoma treatment as well.

Osteosarcoma is the most common 1° bone malignancy in dogs and humans, and progression to metastatic disease and chemoresistance represent the most important treatment hurdles in both species [40,57,58]. Horie *et al*. induced autophagy in a human osteosarcoma cell line using rapamycin, an mTOR inhibitor, while using spautin-1 to prevent the cancer cells from using autophagy as a self-defence mechanism [40]. Spautin-1 alone (100 μM, the same as our high dose) had minimal effect on cell proliferation *in vitro* but significantly reduced cell proliferation in combination with rapamycin, when compared to rapamycin alone [40]. In the same study, a similar effect was seen *in vivo* with shrinkage of xenograft tumours in mice; however, the difference in tumour size when spautin-1 was added as a combination treatment was not significantly different from that of rapamycin alone [40]. These findings are in line with our cell viability results using spautin-1 as a pre-treatment, but our results also indicate that a high dose of spautin-1 was effective on its own. During the *in vivo* portion of Horie *et al*.’s study, no adverse effects were observed in the mice after receiving 40 mg/kg of spautin-1 intraperitoneally, 5 times per week for a period of 4 weeks [40]. A different study stated that spautin-1 induced cardiotoxicity in mice, but there was no data provided to support this statement [43]. In a phase I/II clinical trial for a different autophagy inhibitor, HCQ, used in combination with reduced-dose doxorubicin, 30 dogs with lymphoma had a 93.3% overall response rate, and HCQ-related adverse effects were limited to mild lethargy and gastrointestinal upset [59]. Further *in vivo* studies are required to determine whether spautin-1 is suitable for clinical trials in canine patients with various cancer types.

One study recently showed that a Heat shock protein 90 (Hsp90) inhibitor induced autophagy in 2 metastatic canine osteosarcoma cell lines, one being D17 [60]. Interestingly, the 2 cells lines differed with respect to drug-induced apoptosis, with one cell line exhibiting a significant dose- and time-dependent increase in apoptosis and the other, D17, only minimal apoptosis [60]. The authors suggested that the autophagy induced by the Hsp90 inhibitor was not adequate to enhance apoptosis in the D17 cells [60]. These findings provide evidence that canine osteosarcoma cells perform autophagy and highlight the necessity of using multiple cell lines, based on the observed cell line dependent relationship between autophagy and apoptosis [60].

There were some unexpected expression patterns for several autophagy related proteins between both treatment conditions and cell lines. Doxorubicin consistently reduced cell viability and colony formation in all osteosarcoma cell lines at both doses, as expected. We also anticipated that doxorubicin would increase autophagic activity, and this would be reflected by increased LC3II expression in western blot analysis. This increase was observed in most cell lines after treatment with low dose doxorubicin (lanes 1 vs 4) and in most cell lines with high dose doxorubicin (lanes 4 vs 7), except for the cell lines derived from 1° osteosarcoma (Fig 3). These chemotherapy-naïve cells may be less adept at utilizing autophagy during chemotherapy treatment, compared to the cell lines derived from 2° osteosarcoma which may have previously survived treatment with chemotherapy. Our primary cell line derived from a 2° osteosarcoma had previously been treated with both carboplatin and doxorubicin (S1 Table), but the chemotherapy exposure status of the tumour from which the D17 cell line was generated is unknown.

Protein expression patterns also differed between the non-cancer and the cancer cell lines for pS6RP and beclin-1. The measurement of pS6RP, although it is not a direct substrate of mTOR, can be used to assess mTOR activity [61]. mTOR supresses autophagy, and phosphorylation of S6RP occurs downstream of mTOR activation [61]; therefore, phosphorylation of S6RP is indicative of autophagy suppression and one would expect a decrease in pS6RP expression after doxorubicin treatment. In our study, pS6RP expression increased with increasing doxorubicin concentration in the non-cancer cells but decreased in response to increasing doxorubicin in the osteosarcoma cell lines. This likely reflects the difference in the proliferative rate of normal cells versus neoplastic cells, as doxorubicin targets actively dividing cells. As beclin-1 is required for induction of autophagy, one might expect that treatment with a chemotherapeutic agent would result in increased beclin-1 expression, as was observed in our non-cancer cell lines. However, the opposite effect was observed in the cell lines derived from osteosarcoma, suggesting that autophagy pathways might be altered in these neoplastic cells. Defective autophagy has been implicated in the acceleration of tumour development in some forms of neoplasia [62]. Seeing differences in the expression of autophagy related proteins in our non-cancer versus cancer cells may indicate that abnormal autophagy is involved in the initiation or progression of canine osteosarcoma.

Treating both normal and neoplastic cells with spautin-1 inhibits autophagy, as is indicated by decreased LC3II expression [41,43,45–47,63–73] and increased P62 expression [45,64,66,72] assessed by western blot. In our canine cell lines, we observed the opposite for LC3II; we saw either no change, or increased expression with increasing spautin-1 concentration (Fig 3). For P62, our cancer cell lines produced the expected expression pattern in response to increasing spautin-1; however, the same pattern was not observed in the non-cancer cell lines. Others have similarly reported unexpected protein expression patterns in response to spautin-1 treatment, including a lack of effect on LC3II or P62 [74] and decreased P62 [70]. Expression of both LC3II and P62 are context and cell type specific, they both contribute to cellular processes beyond autophagy, and changes in their expression are not always correlated [61].

Spautin-1, an autophagy inhibitor that increases beclin-1 degradation, typically results in decreased beclin-1 expression using western blot [45,46,66–68,71,75]. Although we observed this pattern in our cell lines derived from 2° osteosarcoma, which were the cells most sensitive to spautin-1 in our study, no effect on beclin-1 was observed in the non-cancer and 1° osteosarcoma cell lines (lanes 1 vs 2 vs 3; Fig 3). Similar to our findings, other investigators did not observe a decrease in beclin-1 expression after spautin-1 treatment [47,76]. These results may indicate that dysregulation of autophagy is relevant to canine appendicular osteosarcoma and that different portions of the pathway may be affected in different cell types (i.e. non-cancerous vs neoplastic and 1° vs 2° osteosarcoma). It is widely accepted that autophagy can enhance both the survival and death of neoplastic cells depending the type of cancer, its genetic profile, and microenvironmental conditions [77]. Therefore, interpreting autophagy related protein expression is not always straightforward, and further investigation into the different patterns between cell types may help elucidate the role of autophagy in pathogenesis and progression of osteosarcoma.

Autophagy related protein expression did not always follow the expected pattern for a given treatment; however, when considering the changes in protein expression, the effects caused by chemotherapy alone must also be considered. High dose doxorubicin diminished the expression of all proteins, including the loading control, most prominently in MDCK cells. This was observed despite both the presence of live cells reflected by the cell viability assays (S1 Fig) and equal protein loading. Some of the inconsistent results observed for high dose doxorubicin may indicate that the cells were unable to perform normal autophagy. Additionally, to assess autophagic carrier flux by western blot, the use of a lysosomal inhibitor is recommended to block degradation of LC3. Using these inhibitors for extended periods of time can lead to nonspecific effects, and maximum exposure time should be under 12 hours [78]. We applied HCQ 6 hours prior to protein collection, which was 18 hours after spautin-1 was removed from the cells. Perhaps the autophagy inhibition induced by spautin-1 was not accurately captured due to the timing of our protein collection, but it did occur and caused significant effects in the cell viability and colony formation assays. Autophagy inhibition could therefore have been responsible for the enhanced chemoresponsiveness seen in our canine osteosarcoma cells.

A definitive explanation of how autophagy contributes to chemoresistance in canine osteosarcoma was not uncovered, but we did observe differences in the expression of autophagy related proteins in response to different treatment conditions, as well as when comparing non-cancer cells to cancer cells and cells derived from 1° osteosarcoma to those derived from 2°. Our results may indicate that cells that have already survived chemotherapy treatment may be better equipped to use autophagy to enhance survival if re-exposed to that drug. Determining which differential response patterns reflect intact versus defective or abnormal autophagy in these canine cell lines will require further investigation. We showed that an autophagy inhibitor, spautin-1, effectively kills canine osteosarcoma cells and reduces their ability to form colonies *in vitro*; and as a pre-treatment, it enhances the effect of doxorubicin in most canine osteosarcoma cell lines. To improve the outcome for dogs diagnosed with appendicular osteosarcoma, new ways to prevent, delay, and/or treat metastatic disease are necessary. Clinically, the addition of spautin-1 to the standard of care could potentially enhance the killing of neoplastic cells and reduce the ability of metastatic osteosarcoma cells to form colonies post-amputation.

## Supporting information

S1 Fig Cell viability dose-response curves for canine cells exposed to doxorubicin. Cell viability measured using a resazurin assay. The solid vertical line denotes the IC50 value, and the dashed vertical lines denote 95% confidence interval (CI) bounds. Error bars denote standard deviation (SD) [CV, coefficient of variation].
(TIFF)

S2 Fig Colony formation dose-response curves for canine cells treated with doxorubicin. Surviving fraction measured using a colony formation assay.
(TIFF)

S3 Fig Cell viability dose-response curves for canine cells exposed to the autophagy inhibitor spautin-1. Cell viability measured using a resazurin assay. The solid vertical line denotes the IC50 value, and the dashed vertical lines denote 95% confidence interval (CI) bounds. Error bars denote standard deviation (SD) [CV, coefficient of variation].
(TIFF)

S4 Fig Expression of autophagy related proteins in canine cells treated with the autophagy inhibitor spautin-1 and doxorubicin. Western blot expression of P62 and LC3 (microtubule‑associated protein light chain 3) in metastatic canine osteosarcoma cells treated with spautin-1 (Low = 15 μM, High = 120 μM) or doxorubicin (IC50) for 24 hours each as a single-agent, or both drugs in combination. Beta actin was used as a loading control. Both in the presence and absence of the lysosomal inhibitor HCQ, LC3II expression is reduced with increasing spautin-1 (1 vs 2 vs 3 and 7 vs 8 vs 9), indicative of autophagy inhibition. Both in the presence and absence of HCQ, doxorubicin increases LC3II expression (1 vs 4 and 7 vs 10), indicative of autophagy induction.
(TIF)

S1 Table Canine cell line origin details. (PDF)

S2 Table Canine cell line experimental details. (PDF)
