# Supplementary figures and images for: The autophagy inhibitor spautin-1, either alone or combined with doxorubicin, decreases cell survival and colony formation in canine appendicular osteosarcoma cells
Source: PLoS One. 2018 Oct 29;13(10):e0206427. doi: 10.1371/journal.pone.0206427 (PMC6205606; doi:10.1371/journal.pone.0206427)

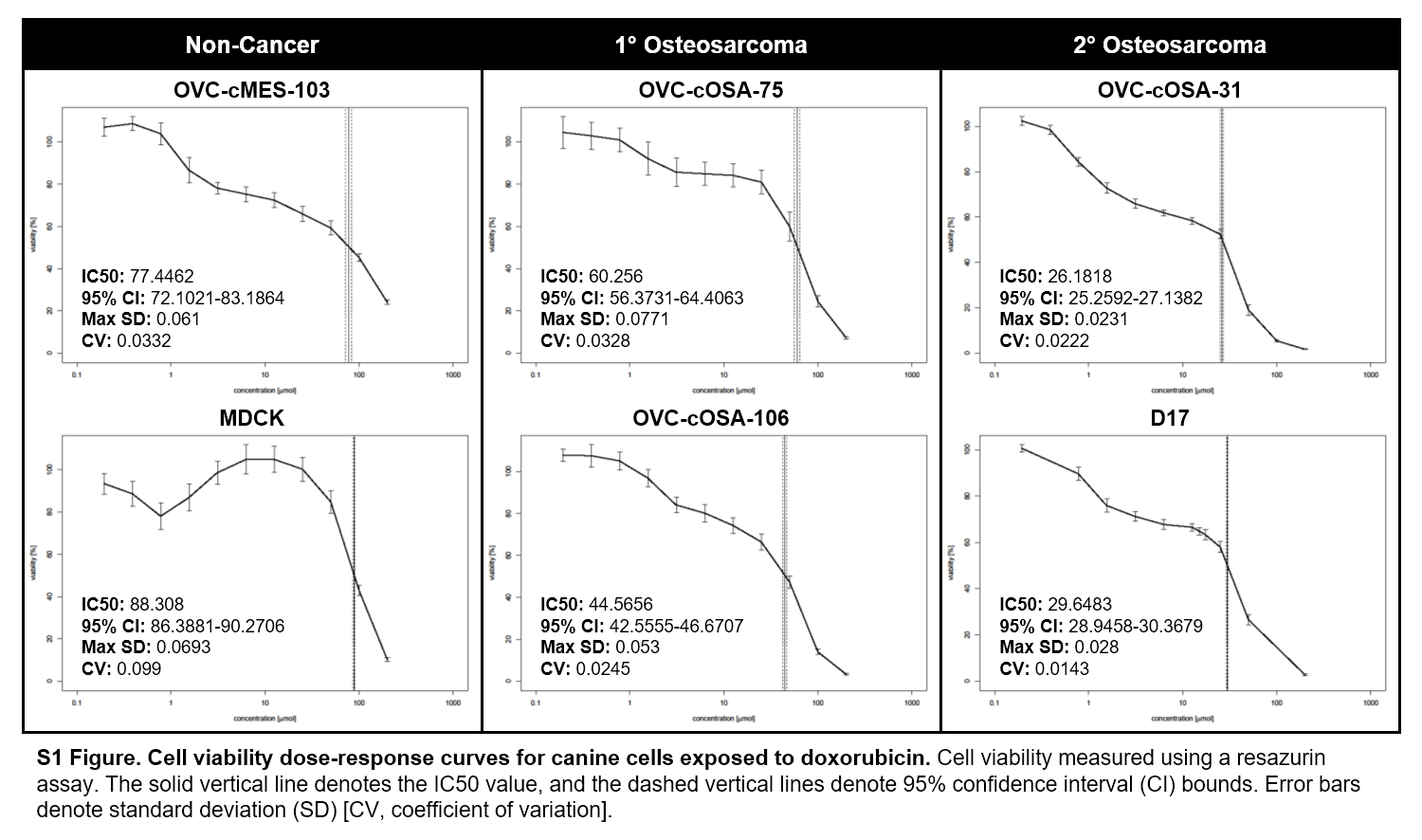

Supplement: S1 Fig — Cell viability measured using a resazurin assay. The solid vertical line denotes the IC50 value, and the dashed vertical lines denote 95% confidence interval (CI) bounds. Error bars denote standard deviation (SD) [CV, coefficient of variation]. (TIFF) [file pone.0206427.s001.tiff]

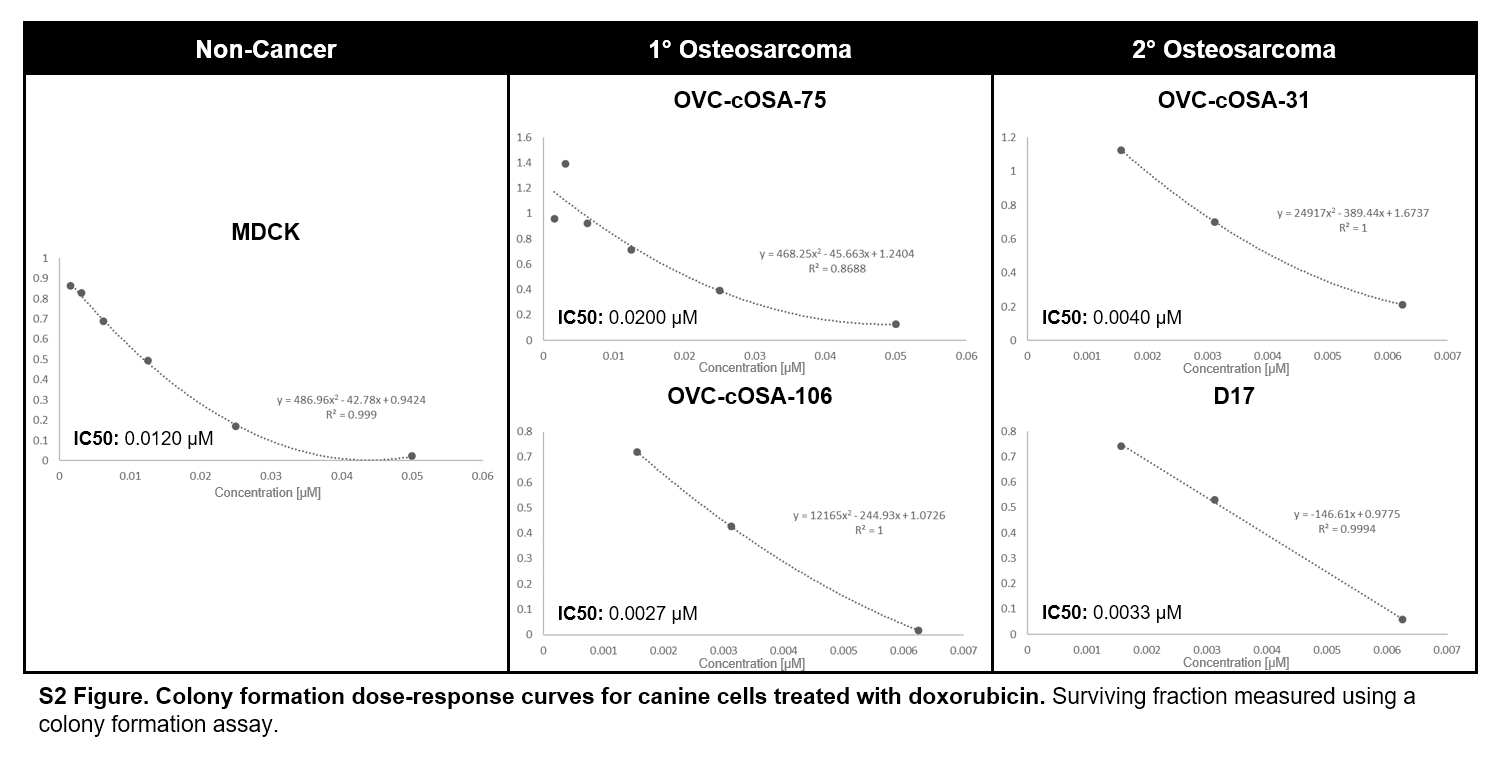

Supplement: S2 Fig — Surviving fraction measured using a colony formation assay. (TIFF) [file pone.0206427.s002.tiff]

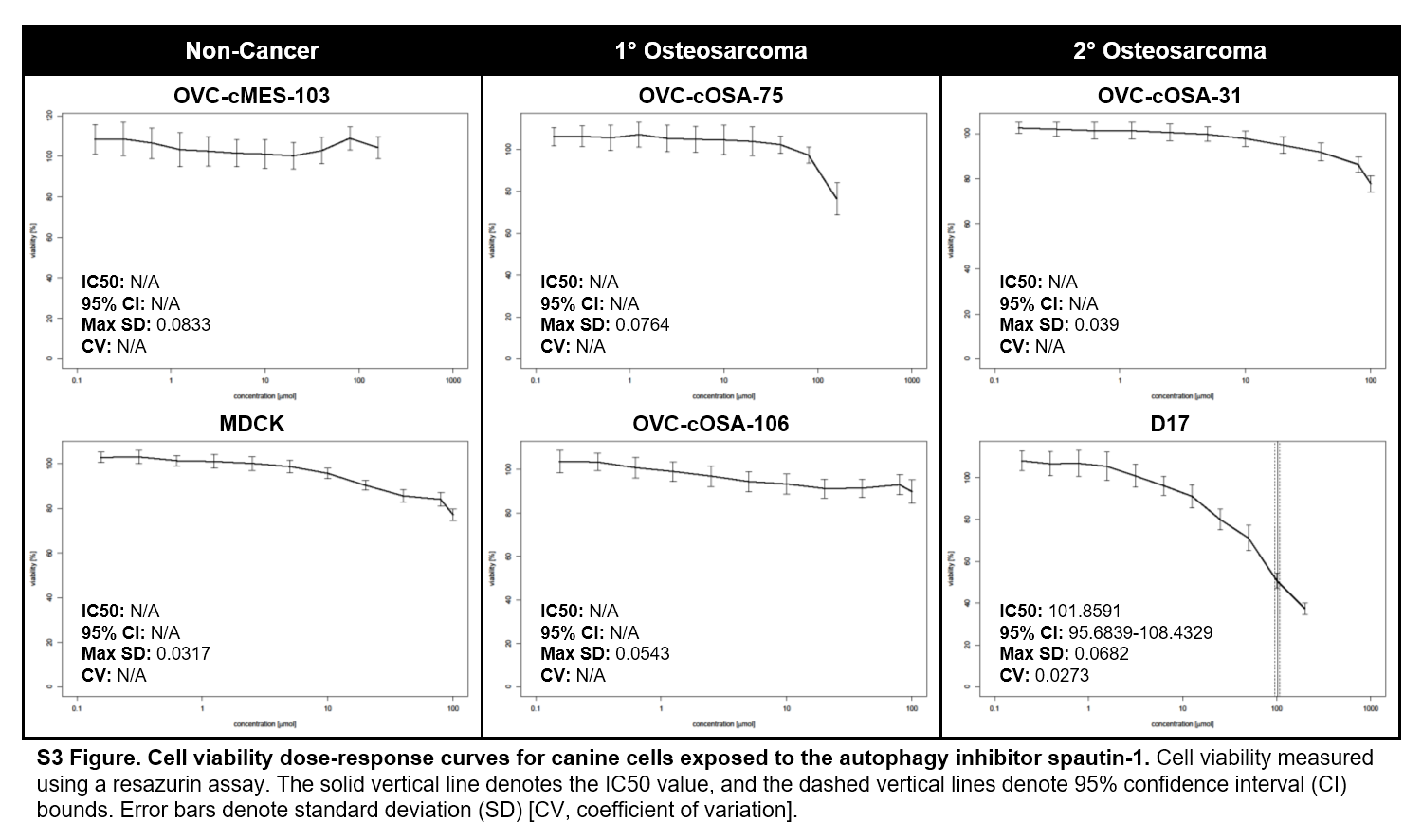

Supplement: S3 Fig — Cell viability measured using a resazurin assay. The solid vertical line denotes the IC50 value, and the dashed vertical lines denote 95% confidence interval (CI) bounds. Error bars denote standard deviation (SD) [CV, coefficient of variation]. (TIFF) [file pone.0206427.s003.tiff]

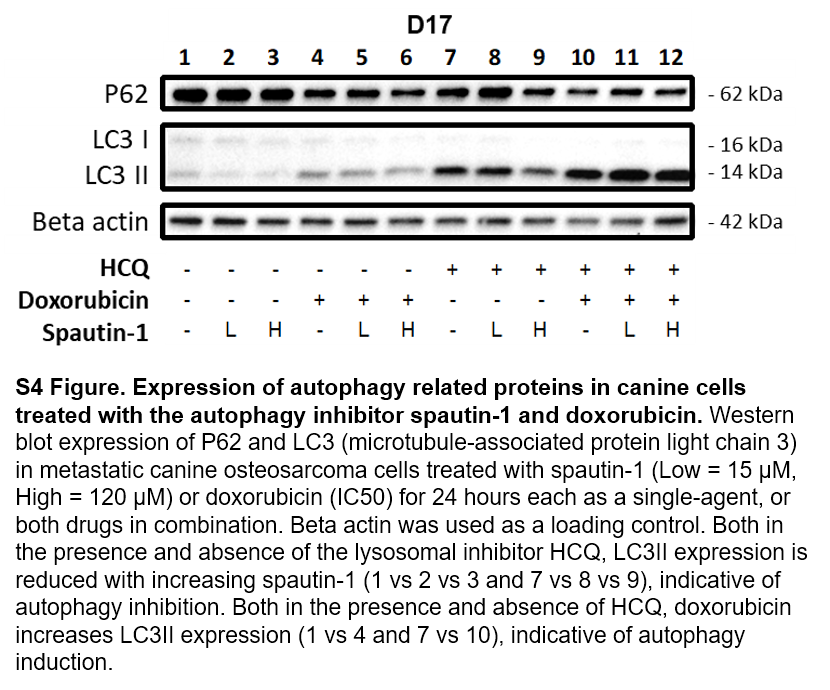

Supplement: S4 Fig — Western blot expression of P62 and LC3 (microtubule‑associated protein light chain 3) in metastatic canine osteosarcoma cells treated with spautin-1 (Low = 15 μM, High = 120 μM) or doxorubicin (IC50) for 24 hours each as a single-agent, or both drugs in combination. Beta actin was used as a loading control. Both in the presence and absence of the lysosomal inhibitor HCQ, LC3II expression is reduced with increasing spautin-1 (1 vs 2 vs 3 and 7 vs 8 vs 9), indicative of autophagy inhibition. Both in the presence and absence of HCQ, doxorubicin increases LC3II expression (1 vs 4 and 7 vs 10), indicative of autophagy induction. (TIF) [file pone.0206427.s004.tif]
