# Supplementary material for: The autophagy inhibitor spautin-1, either alone or combined with doxorubicin, decreases cell survival and colony formation in canine appendicular osteosarcoma cells
Source: PLoS One. 2018 Oct 29;13(10):e0206427. doi: 10.1371/journal.pone.0206427 (PMC6205606; doi:10.1371/journal.pone.0206427)
Supplement: S1 Table — (PDF) [file pone.0206427.s005.pdf]

S1 Table. Canine cell line origin details.

Cell Lines	Sampling Date	Location	Derived Cell Type	Breed	Age (years)	Sex	Chemotherapy Prior to Sampling for Derivation
**Primary**							
**OVC-cMES-103**	2015-10-06	left distal femur	mesenchymal, non-cancer	Greyhound	9.8	SF	no
**OVC-cOSA-31**	2010-05-20	lung	2° osteosarcoma	Bull Mastiff	7.0	CM	yes, 5 carboplatin and 3 doxorubicin
**OVC-cOSA-75**	2013-01-08	left distal tibia	1° osteosarcoma	Greyhound	7.4	SF	no
**OVC-cOSA-106**	2015-12-08	left distal radius	1° osteosarcoma	Doberman	12.2	SF	no
**Commercial**							
**MDCK (ATCC® CCL-34™)**	1958-09	kidney	epithelial, non-cancer	Cocker Spaniel	adult	F	N/A
**D17 (ATCC® CCL-183™)**	1969	lung	2° osteosarcoma	Poodle	11.0	F	unknown

ATCC, American Type Culture Collection; cMES, canine mesenchymal; cOSA, canine osteosarcoma; MDCK, Madin Darby Canine Kidney; OVC, Ontario Veterinary College; ST, survival time
