# Supplementary material for: The autophagy inhibitor spautin-1, either alone or combined with doxorubicin, decreases cell survival and colony formation in canine appendicular osteosarcoma cells
Source: PLoS One. 2018 Oct 29;13(10):e0206427. doi: 10.1371/journal.pone.0206427 (PMC6205606; doi:10.1371/journal.pone.0206427)
Supplement: S2 Table — (PDF) [file pone.0206427.s006.pdf]

S2 Table. Canine cell line experimental details.

	Cell Viability				Colony Formation								
Cell Line	Passage #	Seeding Density (cells/mL)		Doxo IC50 (µM)	Passage #	Days	Seeding Density (cells/well)						Doxo IC50 (µM)
		96-well	6-well				Untreated	5 µM Spautin-1	100 µM Spautin-1	Doxo IC50	5 µM Spautin-1 + Doxo	100 µM Spautin-1 + Doxo	
**OVC-cMES-103**	P8, P9	90000	150000	77	N/A	N/A	N/A	N/A	N/A	N/A	N/A	N/A	N/A
**OVC-cOSA-31**	P10	40000	60000	26	P8, P10	7	300	300	400 & 600	500 or 600	600 & 800 or 700 & 800	700 & 1000 or 800 & 1000	0.0040
**OVC-cOSA-75**	P11	25000	37500	60	P11, P14	9	350 or 450	350	500 & 800 or 450 & 600	600 or 700	600 & 1000 or 700 & 900	800 & 1200 or 700 &900	0.0200
**OVC-cOSA-106**	P6	70000	105000	45	P7, P8	7	1000	1000	2500 & 4000	2000	2500 & 4000	5000 & 8000	0.0027
**D17 (ATCC® CCL-183™)**	N/A	20000	30000	30	N/A	7	250	250 or 300	350 & 500 or 400 & 600	700 or 500	350 & 500 or 500 & 700	700 & 1000	0.0033
**MDCK (ATCC® CCL-34™)**	N/A	67000	100000	88	N/A	6	150	200	400 & 600 or 350 & 500	300	400 & 500 or 350 & 500	500 & 800 or 500 & 700	0.0120

ATCC, American Type Culture Collection; cMES, canine mesenchymal; cOSA, canine osteosarcoma; Doxo, doxorubicin; MDCK, Madin Darby Canine Kidney; OVC, Ontario Veterinary College
